# Site-Specific Integration of Hexagonal Boron Nitride Quantum Emitters on 2D DNA Origami Nanopores

**DOI:** 10.1021/acs.nanolett.4c00673

**Published:** 2024-06-10

**Authors:** Yabin Wang, Ze Yu, Carlas S. Smith, Sabina Caneva

**Affiliations:** † Department of Precision and Microsystems Engineering, Delft University of Technology, Mekelweg 2, 2628 CD, Delft, The Netherlands; ‡ Delft Center for Systems and Control, Delft University of Technology, Mekelweg 2, 2628 CD Delft, Netherlands

**Keywords:** hexagonal boron nitride, nanoparticles, DNA origami, quantum emitters

## Abstract

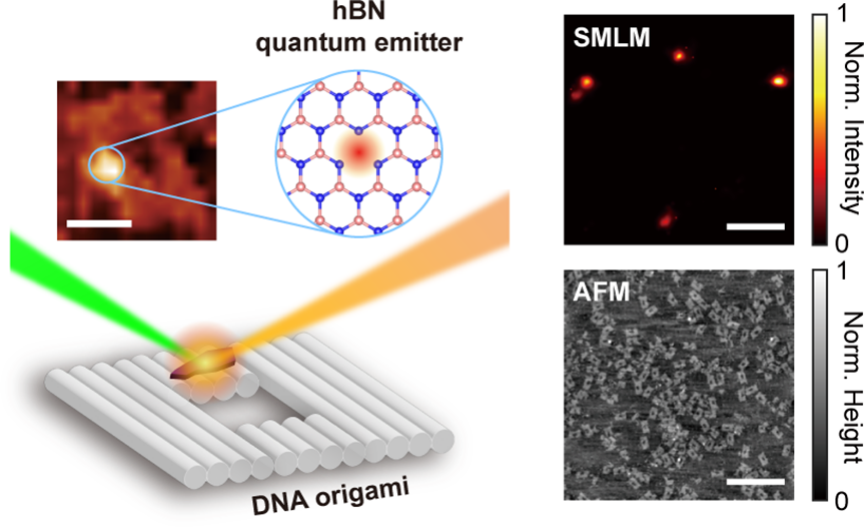

Optical emitters in hexagonal boron nitride (hBN) are promising probes for single-molecule sensing platforms. When engineered in nanoparticle form, they can be integrated as detectors in nanodevices, yet positional control at the nanoscale is lacking. Here we demonstrate the functionalization of DNA origami nanopores with optically active hBN nanoparticles (NPs) with nanometer precision. The NPs are active under three wavelengths of visible illumination and display both stable and blinking emission, enabling their accurate localization by using wide-field optical nanoscopy. Correlative opto-structural characterization reveals deterministic binding of bright, multicolor hBN NPs at the pore rim due to π–π stacking interactions at site-specific locations on the DNA origami. Our work provides a scalable, bottom-up approach toward deterministic assembly of solid-state emitters on arbitrary structural elements based on DNA origami. Such a nanoscale arrangement of optically active components can advance the development of single-molecule platforms, including optical nanopores and nanochannel sensors.

Biosensors play an important role in biomedical research and molecular diagnostics, providing platforms for the analysis, identification, and quantification of biological samples. With the ever-increasing requirement for higher detection accuracy, the demands on biosensor sensitivity are increasing. For example, screening for low-abundance proteins in human plasma is crucial for the identification of disease-specific biomarkers.^1^ There is therefore an urgent need to develop techniques that operate at the ultimate sensitivity, i.e., at the single-molecule level.^2^ Unlike traditional ensemble techniques, which sample large collection of molecules simultaneously, thereby blurring out the details of individual components, single-molecule techniques narrow the focus to the single molecule level.^3^ These techniques can be based on the detection of optical,^4^ electrical, or mechanical signatures^5,6^ to determine the interaction between the sensor and the target molecule. Among single-molecule detection technologies, nanopore sensors are emerging as ground-breaking tools that enable single-molecule analysis at sub-nm resolution.^7^ Ionic current nanopore measurements have been extremely successful in DNA and peptide sequencing, enzymology and protein analysis, yet scaling this technique requires relatively complex electrical isolation and amplifier setups.^8^ Optical nanopore detection, on the other hand, is receiving increasing attention as it enables parallel detection capability through wide-field imaging of high-density nanopore arrays while retaining single-molecule resolution.^9^ By tracking changes in optical signals generated by the interaction between target molecules and nanopores, real-time screening of samples can be performed, providing statistically significant data sets from thousands of individual nanopores within the field-of-view. Optical nanopore sensing can be implemented in various configurations, including through the detection of fluorophores,^10−12^ variations in fluorescence indicator dye fluxes,^13,14^ plasmonic nanostructure enhanced transmission^15,16^ and surface-enhanced Raman scattering.^17^ While these platforms are based on different physical principles, they require the precise location of robust optical sensors on the nanoscale to enhance the interaction with the analyte of interest.

Optical probes typically consist of fluorescent dyes, yet novel solid-state materials are emerging that show promising properties as nanoscale sensors and can be readily integrated in nanopore platforms. Specifically, quantum emitters (QE) formed by lattice defects in two-dimensional (2D) hexagonal boron nitride (hBN) have gained significant attention in recent years due to their desirable combination of optical, chemical and mechanical properties.^18^ hBN is a van der Waals material in which alternating boron (B) and nitrogen (N) atoms are arranged in a honeycomb lattice by strong covalent bonds. Atomic hBN layers are held together by weak van der Waals forces and can thus be easily exfoliated to atomically thin sheets. Unlike graphene, hBN is an electrical insulator, exhibiting a wide bandgap of ∼6 eV,^19^ and is therefore optically transparent in the visible range. In analogy to diamond, however, the hBN lattice can host a diverse spectrum of crystalline defects which act as color centers featuring high brightness (4000 kcts/s),^20^ long lifetimes (∼3 ns),^21^ high quantum efficiency (87%),^22^ emission in the visible,^23^ room-temperature operation,^24^ stability in liquid environments,^25^ and biocompatibility.^26^ This combination of properties has propelled hBN to the forefront of research in nanophotonics, super-resolution imaging, and biolabeling. Optically active defects in hBN can generated by various means including postgrowth annealing,^27^ wet chemical etching,^28^ electron and ion beam irradiation,^17,29^ and deliberate introduction of carbon impurities during growth.^30^ Progress has also been made to deterministically localize hBN QE by generating defect arrays with AFM tips,^31^ femtosecond lasers,^32^ and focused ion/electron beams,^33,34^ as well as via strain patterning.^35^ While optically active porelike structures can be fabricated in hBN membranes, the control over the density and distribution of the QE around the pore has remained elusive. Here we address the site-specific integration of the hBN QE in nanopore structures by spatially engineering the binding sites of fluorescent hBN nanoparticles (NPs) on DNA origami nanopores.

As a flexible and programmable structural platform with precise site addressability, DNA origami is often used as a breadboard on which to attach a variety of functional molecules or nanoparticles including metal nanoparticles,^36,37^ proteins^38^ and fluorophores^39^ with single base resolution. Functionalization can be achieved through hybridization of short complementary DNA oligonucleotides between the component to be added and single-stranded DNA (ssDNA) integrated into the origami structure.

Due to their hexagonal crystal structure, 2D materials can enable facile integration with DNA via π–π stacking interactions.^40^ Several studies investigated ssDNA–graphene systems by exploiting the strong noncovalent DNA–2D material interaction.^41−44^ This interaction was, for example, used to functionalize a DNA origami nanobreadboard featuring ssDNA “hooks”, with carbon nanotubes as electronic components.^43^ Recently, the ssDNA–graphene system was utilized to immobilize dsDNA segments on graphene for DNA–protein interaction studies.^45^ While DNA–graphene platforms are gaining traction, DNA–hBN systems have been less explored and, to date, have primarily been limited to computational studies.^46,47^ Recent experimental work demonstrated the interaction of unlabeled dsDNA with hBN nanochannels via interferometric scattering microscopy,^48^ as well as the diffusion behavior of labeled ssDNA on both pristine and defective hBN surfaces via fluorescence nanoscopy.^49^ The affinity of ssDNA to hBN forms the basis for building a novel class of hybrid devices that harness the exquisite structural programmability of DNA origami with the unique optical properties of hBN QEs.

Here, we present a simple and scalable way to optically functionalize DNA-based nanopores with hBN NPs. We employ a cryogenic-pretreatment liquid phase exfoliation (LPE) method to produce hBN NPs featuring crystal defects^50^ and subsequently use single molecule localization microscopy (SMLM) to investigate the optical properties of the QEs under three excitation wavelengths (473, 532, and 640 nm). To demonstrate site-specific positioning of the QEs at the nanoscale, we designed and assembled a square DNA origami nanopore featuring an attachment site composed of 19 ssDNA next to the edge of the pore. We leverage the π–π stacking interaction between the hBN basal plane and the DNA bases to spatially control the adsorption between hBN NPs and ssDNA at the nanoscale. Specifically, using correlative optical and structural analysis based on SMLM and atomic force microscopy (AFM), supported by molecular dynamics (MD) simulations, we demonstrate the first optically active hBN-DNA origami nanopores. The precise localization of hBN QEs can form the basis for fluorescence-based sensing and sequencing of biomolecules via, e.g., Förster resonance energy transfer (FRET) in optically active nanopores.^51,52^

To produce hBN NPs with optical emitters, we utilized a cryogenic pretreatment followed by a LPE step. The methodology is illustrated in Figure 1a. This approach introduces lattice defects in the hBN lattice through thermal shock, followed by the breaking of bulk crystals into NPs via ultrasonication in a water bath. The hBN NPs are separated from the solution by subsequent centrifugation and filtration (see section 1 in the Supporting Information (SI) for details about the fabrication).

**Figure 1:**
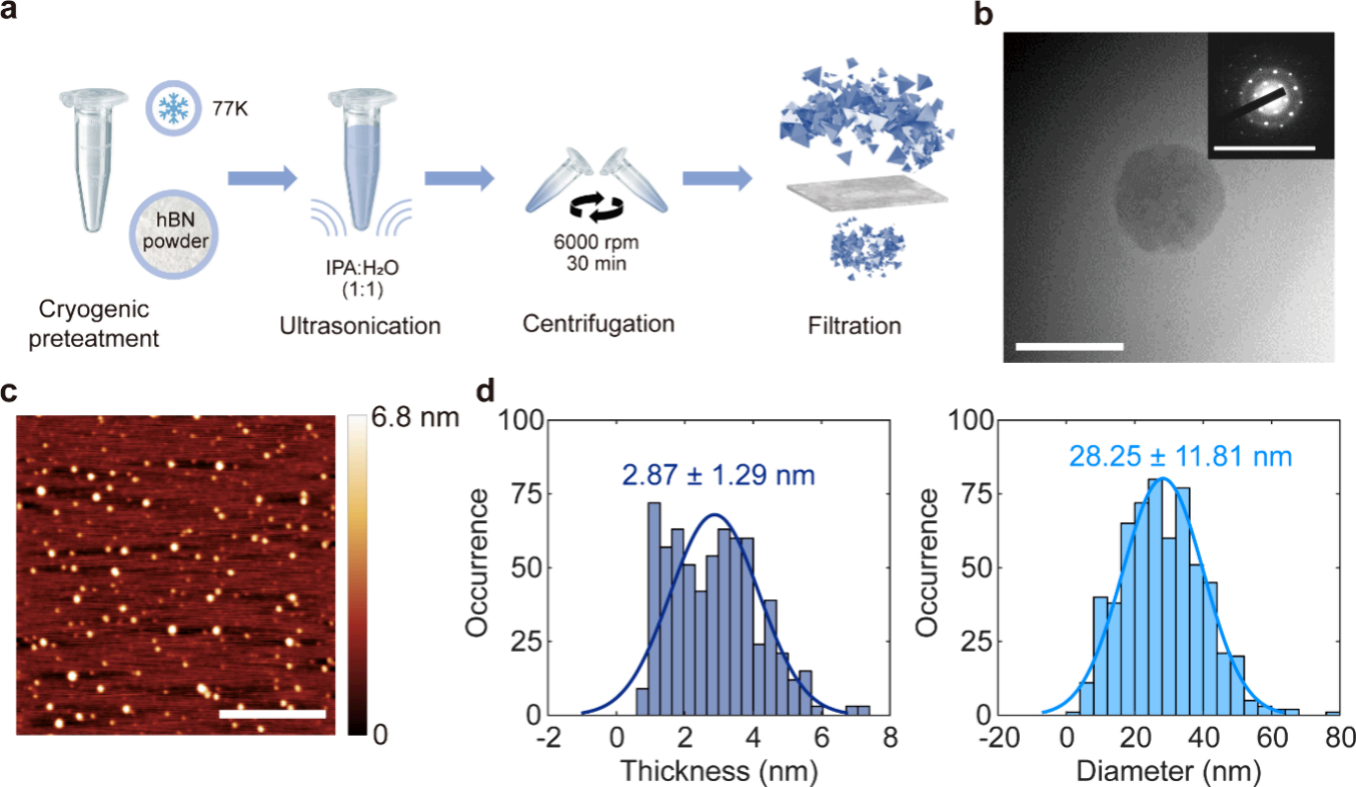
hBN NP production and structural characterization. (a) Schematic of the cryogenic pretreated liquid phase exfoliation of hBN NPs. The hBN powder was immersed in liquid nitrogen for 1 h and then dispersed into room-temperature IPA/H_2_O to generate cracks in the bulk material with thermal shock. The solution was ultrasonicated for 4 h to break the bulk material. To separate the NPs from the dispersions, the resultant solution was centrifuged at 6000 rpm for 30 min and filtered through a filter with a pore size of 100 nm. (b) TEM image of a representative hBN NP (scale bar = 100 nm). Inset: Electron diffraction pattern of the corresponding NP (scale bar = 1/0.03 nm^–1^). (c) AFM image of the diluted hBN NPs on a mica substrate (scale bar = 500 nm). (d) Distribution of the thickness (left) and diameter (right) of the NPs acquired from 600 NPs.

For detailed structural analysis, we imaged the hBN NPs using transmission electron microscopy (TEM). Figure 1b shows a representative image of a NP with diameter smaller than 100 nm (inset) as well as its diffraction pattern, which exhibits the characteristics of hexagonal symmetry expected for monolayer hBN with a lattice parameter of 2.54 Å.

We further characterize the size distribution of hBN NPs with AFM, as shown in Figure 1c. The measured topography reveals a broad diameter distribution of the NPs, ranging from 10 to 100 nm. Statistical analysis of the AFM results from 600 NPs in Figure 1d yields an average diameter of 28.25 nm and a thickness of 2.87 nm, thus, significantly smaller than the original crystal powder with a 100 nm diameter (see section 2 in the SI for details about the AFM measurement).

To investigate the optical properties of the QEs in hBN NPs, we characterize the sample in an inverted widefield microscope equipped with three lasers (473, 532, and 640 nm; see section 4 in the SI for the optical setup). Figure 2a illustrates the wide-field image in the three channels. To characterize the photodynamics of the QEs, their emission intensity traces are analyzed. The emitters can be classified into two types: stable and blinking. Representative traces of each type are shown in Figures 2b,c and S1. During 1 min of exposure, stable emitters continuously displayed a consistent high intensity level. In contrast, blinking emitters intermittently switch from high intensity to low intensity levels. The different photodynamic responses suggest differences in the hBN defect composition and their interaction with the external environment, which is in agreement with previous reports.^53,54^

**Figure 2:**
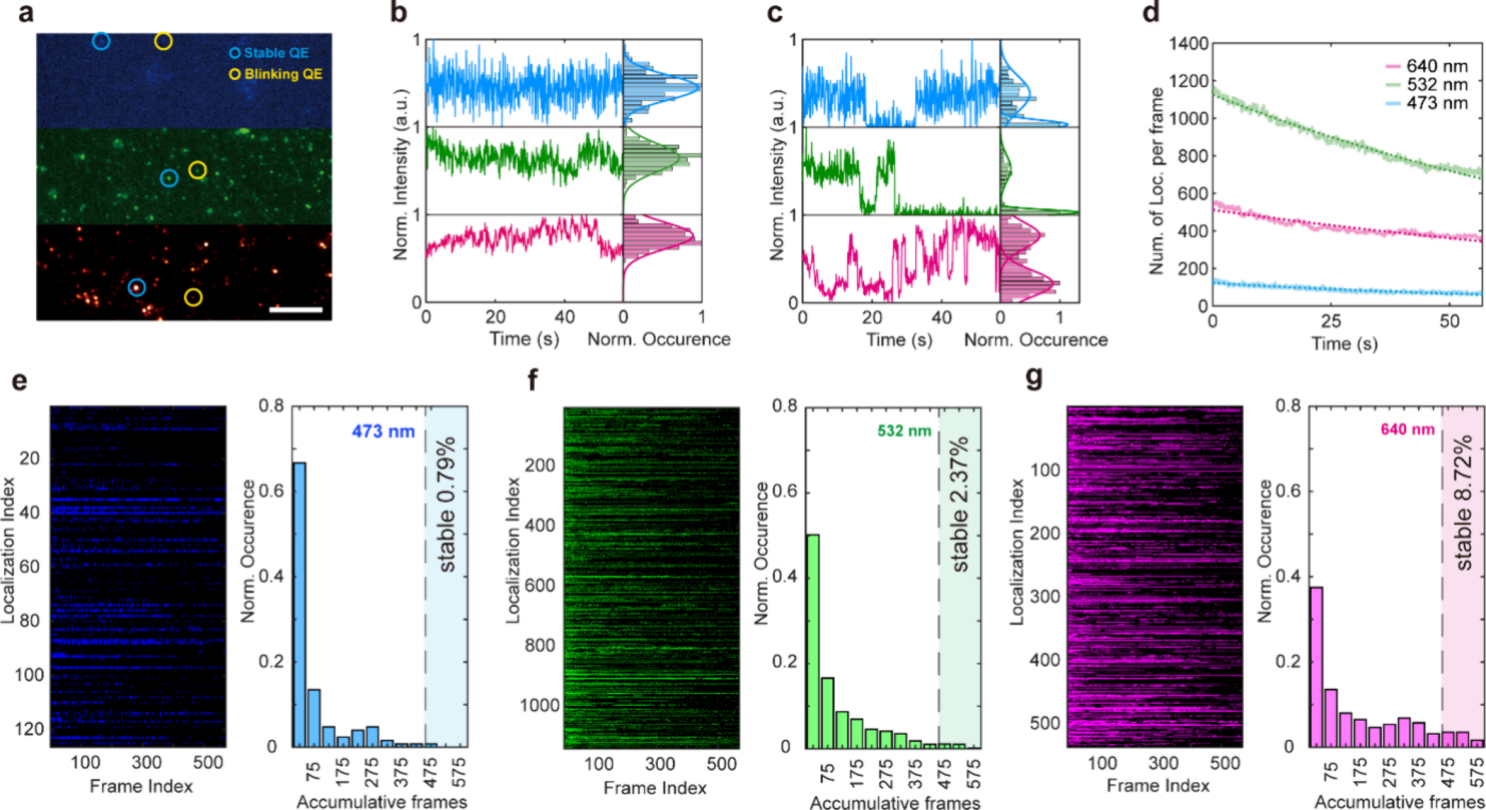
Optical characterization of QEs in hBN NPs. (a) Widefield image of the QEs excited by three different channels (excitation wavelengths: 473, 532, and 640 nm, power = 100 mW; scale bar = 10 μm), two representative quantum emitters with different photon dynamics are circled in each channel: (b) intensity–time trace of the stable quantum emitters and (c) intensity–time trace of the blinking quantum emitters. (d) Temporal evolution of the number of localizations per frames. (e–g) Photon dynamics of the hBN QEs excited by three different channels (excitation wavelengths: 473, 532, and 640 nm). Left panel: Visualization of the QEs ON (bright color) and OFF (black) along the time series. Right panel: histogram of the “ON” frame amount, if the amount of the “ON” frame is over 80% of the whole time series (48 s), we define it as a stable QE (see Figure S2 for detailed classification of the QEs).

To characterize the impact of the excitation wavelengths on the behavior of quantum emitters, we trace the number of localizations per frame within a defined sample area (56 μm × 56 μm) during 60 s exposure time. Under 640, 532, and 473 nm excitation with the same laser power of 100 mW, the traces demonstrate exponential decrease with a characteristic time of 143.85, 112.08, and 80.8776 s, respectively, which we attribute to photobleaching. The fraction of the stable quantum emitters, as shown in Figure 2e–g, i.e., emitters with >80% “on” time over a period of 60 s, is 8.72%, 2.37%, and 0.79% under red, green, and blue excitation. Both the photobleaching time and the proportion of stable emitters indicate that quantum emitters are more stable at higher wavelength excitations. The total number of localizations indicates that the majority of quantum emitters are activated by a 532 nm laser, while 473 nm excitation activates only a small fraction (11.08%) of the QEs. However, due to bleaching and the order of the measurements, first exposure to 640 nm, then 532 nm and last with 473 nm, the localization numbers in the following two channels (1137 localizations and 126 localizations under 532 and 473 nm excitation, respectively) are likely underestimated. The high number of localizations proves the efficiency of cryogenic LPE in producing QEs.

The blinking behavior of the QEs enables super-resolution imaging. We use SMLM to determine the localization of QEs from a sample area consisting of the three merged channels.^34,55^Figure 3a illustrates the wide-field tricolor image where the smeared, diffraction-limited spots prevent a clear localization of the defects. Figure 3b compares the diffraction-limited zoomed-in region of QEs as-recorded (top) and the same region after SMLM reconstruction (bottom). We find that some QEs are activated exclusively by a single excitation wavelength, while others respond to two or all three wavelengths. Previous research on hBN flakes has revealed that the nature of the crystal defects gives rise to a wide range of energy transitions from which the emission originates,^54^ which suggests that various defect types are present in the hBN NPs. Furthermore, as shown in Figure 3c, we use photoluminescence (PL) spectroscopy to characterize the spectral properties of the QEs. Excluding the first peak at 554.3 nm, which corresponds to the *E*_2G_ Raman mode of hBN, the peaks of the emission spectrum widely distribute in the 561 nm (2.21 eV) to 639 nm (1.94 eV) range, which is consistent with our fluorescence microscopy results and previous research.^56^ While on one hand controlling the property of the quantum emitters in these NPs is challenging, on the other hand, they offer optical probes that can be used to multiplex biomolecule sensing in various wavelength ranges. In particular, this feature could be leveraged for multicolor FRET measurements, where two or more differently labeled analytes could be detected by the same hBN NPs, which contains probes with distinct spectral properties.

**Figure 3:**
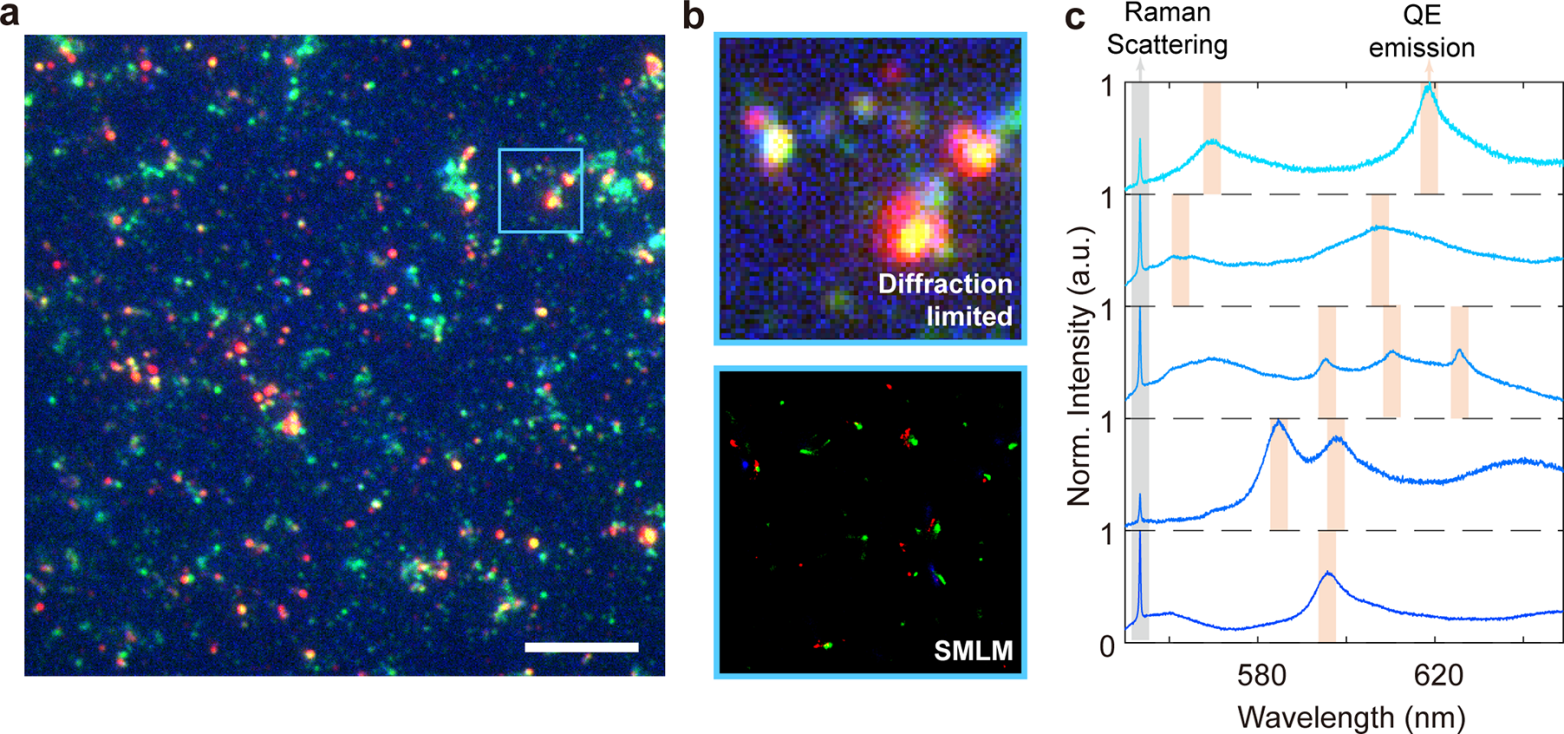
Spatial and spectral analysis for the quantum emitters in hBN NPs. (a) Widefield merged 3-channel fluorescence microscopy image of the sample (scale bar = 10 μm). (b) Top: zoomed-in image of the highlighted blue square area in (a); the smeared patterns due to the diffraction limit hinder the precise localization for the quantum emitters. Bottom: SMLM reconstructed an image of the same area in the top area with 570 frames in three different channels using the ImageJ plugin ThunderSTORM. (c) Five representative emission spectra of the quantum emitters in the same sample under 514.5 nm excitation.

We demonstrate deterministic, site-specific integration of hBN NPs on DNA origami by designing a two-dimensional monolayer DNA origami nanopore structure with attachment sites near the pore, as depicted in Figure 4a. This structure is 80 nm in length and 60 nm in width, with a central square cavity of 20 nm. To facilitate the binding of hBN NPs onto the origami structure, we introduced 19 single-stranded DNA oligos on one side of the pore, each composed of 30 adenine (A) bases.

**Figure 4:**
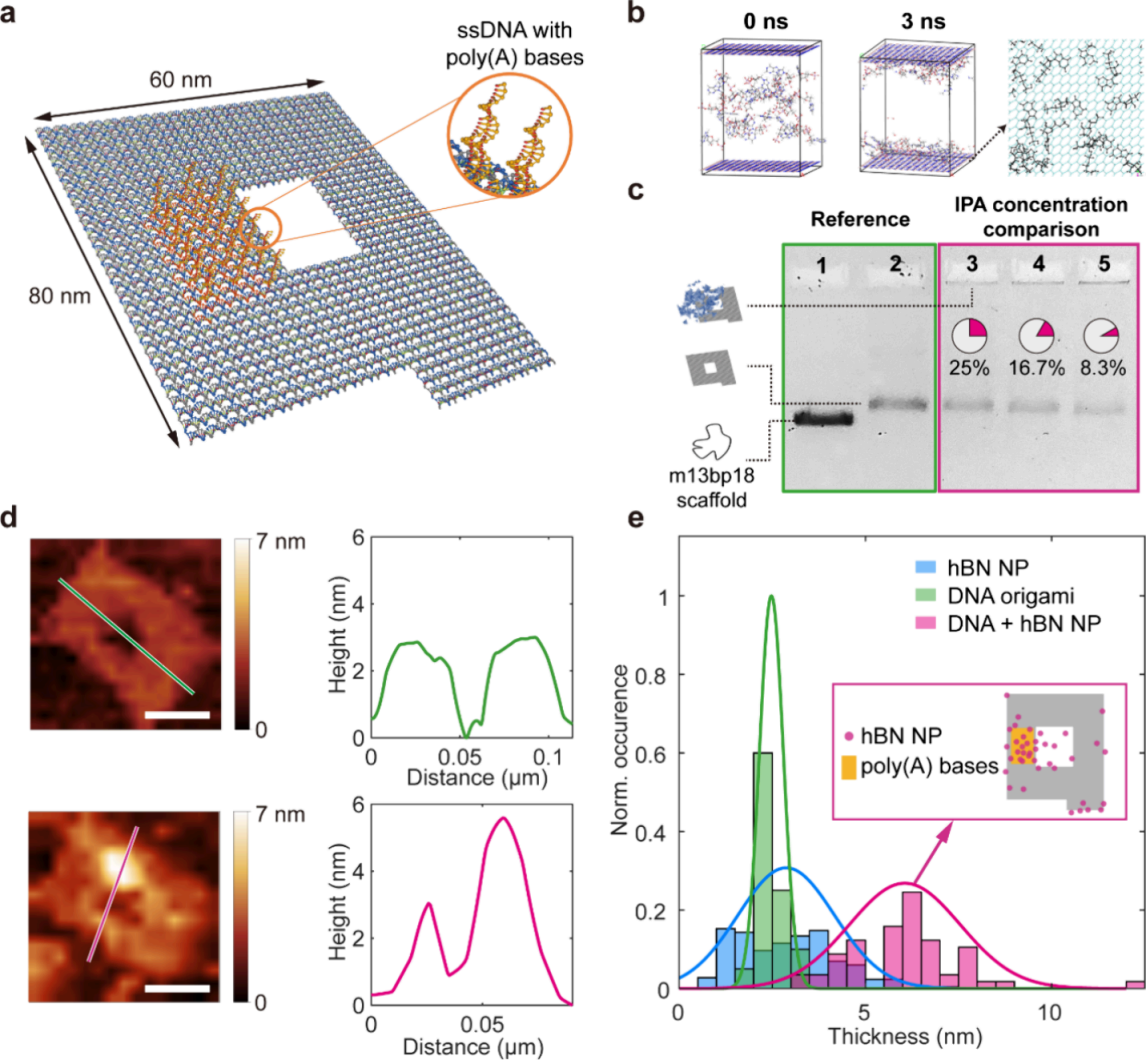
Characterization of hybrid hBN–NPs/DNA origami nanopores. (a) Schematic illustration of the DNA origami nanopore: the orange strands indicate the position of the 19 “sticky” ssDNA composed of 30 A bases each. (b) MD simulation of the interaction between DNA bases and the hBN flakes in H_2_O/IPA. Here we only show the result of adenine bases, and the IPA and H_2_O molecules are removed for clarity. After 3 ns, the DNA bases are attached to the hBN surface. The color of the top view is changed for better visualization. (c) Agarose gel electrophoresis of DNA origami and DNA origami–hBN NPs complexes: 1: m13bp18 scaffold, 2: DNA origami, 3: DNA origami + hBN NPs dispersed in H_2_O/IPA (IPA volume ratio = 25%), 4: DNA origami + hBN NPs dispersed in H_2_O/IPA (IPA volume ratio 16.7%), 5: DNA origami + hBN NPs dispersed in H_2_O/IPA (IPA volume ratio 8.3%). (d) Representative AFM measurements and height profiles along the DNA origami surface for (top) the bare DNA origami nanopore and (bottom) hBN NPs-functionalized DNA nanopore. (Scale bar: 40 nm) (e) Histogram of the thickness of the samples: the mean heights of hBN NPs, DNA origami, and the complex are 2.87, 2.48, and 6.05 nm, respectively. The inset map indicates the distribution of the hBN NPs on DNA origami.

We first analyze the interaction between hBN and nucleotides through MD simulations. We performed MD simulations of different nucleobases adsorbing to hBN flakes. To closely match the experimental conditions, in which hBN NPs/flakes are dispersed in IPA/H_2_O, both IPA and H_2_O molecules were added to the system and we subsequently introduced the same number of different types of nucleobases to each simulation. Overall, as shown in Figure 4b, the configurations in the initial state (0 ns) and after 3 ns demonstrate the preferential adsorption behavior of nucleobases on hBN surfaces (see section 6 in the SI for the setting of the MD simulation and Figure S3 for the DNA base distribution after 3 ns and the potential energy distribution).

Following the assembly and purification of DNA origami, we used agarose gel electrophoresis to investigate the interaction between DNA origami and hBN NPs. We mix the DNA origami and the hBN NPs with various solvents using the m13bp18 scaffold (7249 bases) as reference (see section 7 in the SI for details about the gel electrophoresis). The electrophoresis results are shown in Figure 4c. It can be observed that with the same DNA origami concentration and hBN nanoparticle concentration for each lane, the intensity of the bands formed by the hBN–DNA complex decreased significantly with the increase in the volume fraction of IPA in the solvent, indicating that the yield of the hybrid structure is largely dependent on the solvent composition, which plays an important role in controlling the binding affinity of hBN to DNA.

To further investigate the effect of solvent on the adsorption between ssDNA and hBN, we image the adsorption of Cy3B-labeled ssDNA with hBN flakes in different solvents using TIRF microscopy. The results are consistent with the gel electrophoresis experiment, in which higher concentrations of IPA weaken the adsorption efficiency and thus reduce the yield of the hBN NPs bound to origami (Figures S4–S6).

To further prove that we indeed obtain a hybrid structure of hBN NPs bound to DNA origami, we carry out AFM imaging to characterize the topography of both the bare DNA origami and the DNA origami–hBN NPs (see section 2 in the SI for details about the AFM measurement). In Figure 4d, we present two representative measurements depicting the height profiles of the samples. The adsorption of hBN NPs on the DNA origami plate (bottom image) disrupts the symmetrical profile of the bare DNA origami (top image). By measuring 40 samples, the statistical results reveal that the mean thickness of the hBN NPs, DNA origami, and DNA origami–hBN NPs complex is 2.87, 2.48, and 5.66 nm, respectively (raw images in Figures S7 and S8). Leveraging the short tail of the DNA origami plate, we examine the relative positioning of the hBN NPs on DNA origami, as shown in Figure 4e. The majority of binding occurs at the prescribed ssDNAs “sticky area” near the inner pore rim, however, the attachment on the outer edges of the DNA origami is also observed. We attribute adsorption at the edges of the plate to the “free ends” of the DNA origami, which we left to be ssDNA to prevent the aggregation of the DNA origamis^57^ (see Figure S9 for the details about the aggregation due to the edge staples).

We demonstrate that the hBN NPs are still optically active after integration on the DNA origami by performing correlative fluorescence microscopy and AFM. Here we introduce polystyrene microspheres (15 μm in diameter) on the surface as the references to correlate the fluorescence microscopy and AFM (details in SI, Figures S10 and S11). Based on the previous experiment, to avoid areas of high hBN aggregation, as shown in Figure S12, here we mix the DNA origami with hBN NPs with a volume ratio of 2:1, which leads to a relatively low ratio of organic solvent but enough hBN for the integration. The SMLM reconstruction under 532 nm excitation and AFM scan of the same area are shown in Figure 5a,b. As shown in Figure 5c, after the calibration, the correlative results allow us to localize the hBN QE on the DNA origami. We also notice that some hBN nanoparticles are not fluorescent or too dim to be detected, which could be attributed to the insufficient or non-optically active defects in the crystal structure under 532 nm illumination. Figure S13 also shows the intensity traces of the integrated hBN quantum emitters, which are similar to the “free” (unbound) hBN quantum emitters, showing the feasibility of integrating hBN quantum emitters into biological systems without affecting their optical properties.

**Figure 5:**
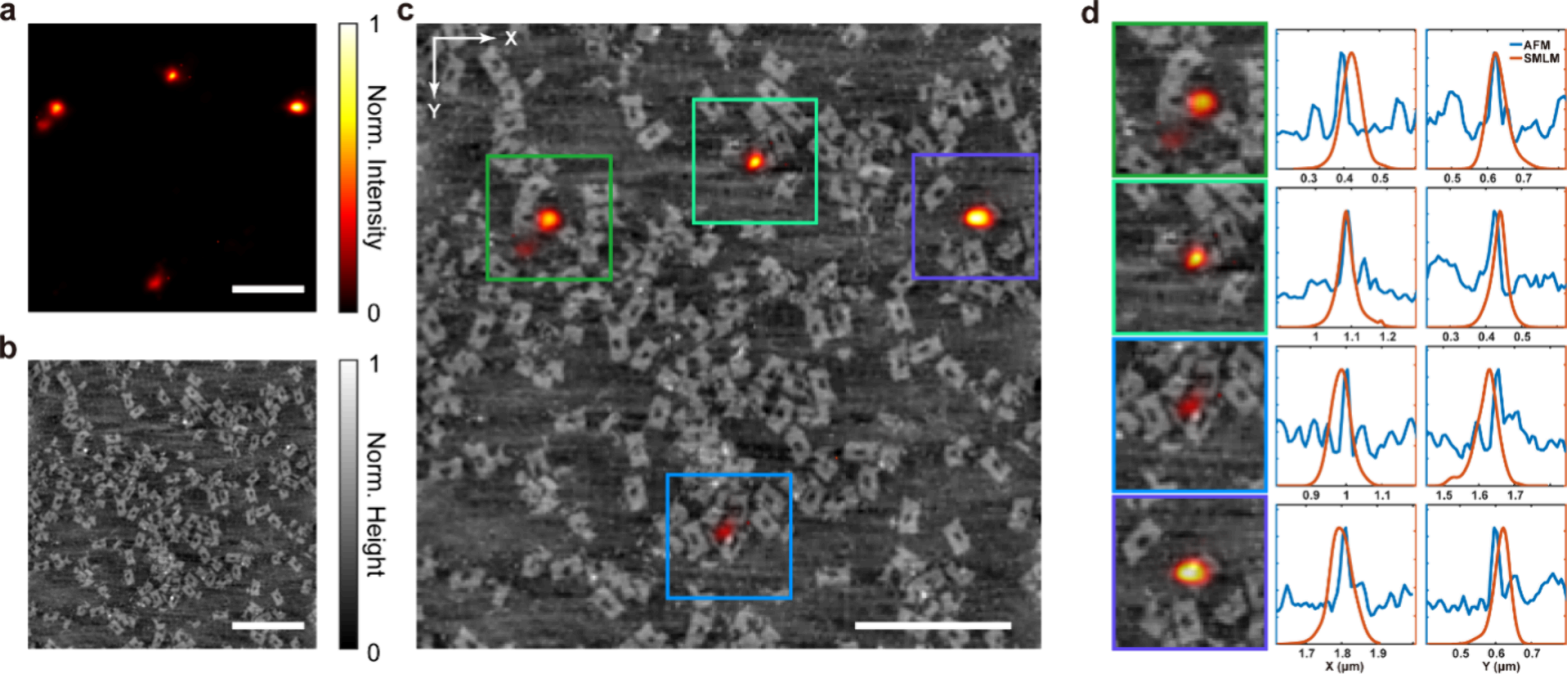
Correlative opto-structural characterization of hybrid hBN QE/DNA origami nanopores. (a) Reconstructed SMLM image of hybrid hBN QE/DNA origami nanopores on the polylysine coated coverslip under 532 nm excitation, using the ImageJ plugin ThunderSTORM (scale bar = 500 nm). (b) AFM measurement of the same area (scale bar = 500 nm). (c) Correlative AFM and SMLM measurements after angle and translocation alignment (scale bar = 500 nm). (d) Representative correlative measurements of hBN QE on DNA origami nanopores and the corresponding SMLM/AFM profiles of the NP along the *x* and *y* directions.

In this work we report the assembly of a novel class of optical nanopores composed of fluorescent hBN NPs integrated in the rim of DNA origami nanopores. The hBN NPs with an average lateral size <30 nm and thickness of <3 nm are produced by cryogenic pretreated liquid-phase exfoliation and host crystal defects that are activated by red, green, and blue laser excitation. The PL signatures of these QE exhibit different spectral and temporal properties, suggesting the presence of various defects in the hBN NPs that respond differently to the environment. Both stable and blinking emitters are generated and SMLM can therefore be employed to successfully localize and correlate distinct QEs across different channels. We achieve deterministic placement of the optical emitters by leveraging the π–π stacking interactions between the hBN basal plane and the aromatic ring of the DNA bases, which is supported by MD simulations. Specifically, by preparing DNA origami platelets featuring a central nanopore with ssDNA handles near the rim, we demonstrate that hBN NPs preferentially bind to the handles. Opto-structural characterization through AFM imaging and single-molecule fluorescence microscopy confirms the site-specific integration of these solid-state optical probes with structural DNA molecules. The further modulation of QEs can be achieved by changing the pH of the buffer,^58^ the buffer composition (e.g., organic solvent),^25^ and the substrate of the sample.^59^ This scalable generation of QE-functionalized nanopores can be implemented for single-molecule biosensing and sequencing schemes based on high-parallel optical readouts. Multi-color hBN NPs offer the potential for multiplexed detection by exciting nanopores with multiple laser wavelengths without increasing the number of integrated probes. They could thereby enable high-resolution and high-throughput screening of biological samples for application in nanofluidics and molecular diagnostics.

## Supporting Information Available

1. Production of hBN nanoparticles; 2. AFM sample preparation and imaging; 3. TEM sample preparation; 4. Microscopy setup; 5. DNA origami design and assembly; 6. MD simulation parameters; 7. Agarose gel electrophoresis; 8. Fluorescent intensity traces under three illumination wavelengths; 9. QE dynamics (stable/blinking) classification; 10. MD simulation results; 11. Sample preparation for the investigation of the interaction between ssDNA and hBN flakes in different solvent environments; 12. Schematic illustration for the fluorescence experiment; 13. Solvent influence on the interaction between ssDNA and hBN; 14. AFM images of DNA origami nanopores before hBN NP integration; 15. AFM images of DNA origami nanopores after hBN NP integration; 16. Aggregation due to the edge staples of the DNA origami; 17. Correlative FM-AFM sample preparation; 18. Correlative FM-AFM based on fiducial beads; 19. hBN aggregation AFM results; 20. Intensity of the integrated quantum emitters on the DNA origami (PDF)
