# Supplementary material for: Site-Specific Integration of Hexagonal Boron Nitride Quantum Emitters on 2D DNA Origami Nanopores
Source: Nano Lett. 2024 Jun 10;24(28):8510–7. doi: 10.1021/acs.nanolett.4c00673 (PMC11261624; doi:10.1021/acs.nanolett.4c00673)
Supplement: Supplementary file 1 — nl4c00673_si_001.pdf [file nl4c00673_si_001.pdf]

Supporting Information: Site-specific Integration of Hexagonal Boron Nitride Quantum Emitters on 2D DNA Origami Nanopores

Yabin Wang,[1,2] Ze Yu,[1] Carlas S. Smith,[2] Sabina Caneva*[1]*

[1] Department of Precision and Microsystems Engineering, Delft University of Technology, Mekelweg 2, 2628 CD, Delft, The Netherlands

[2] Delft Center for Systems and Control, Delft University of Technology, Mekelweg 2, 2628 CD Delft, Netherlands

Content

1. Production of hBN nanoparticles 2
2. AFM sample preparation and imaging 2
3. TEM sample preparation 2
4. Microscopy setup 2
5. DNA origami design and assembly 2
6. MD simulation parameters 3
7. Agarose gel electrophoresis 3
8. Fluorescent intensity traces under three illumination wavelengths 4
9. QE dynamics (stable/blinking) Classification 5
10. MD simulation results 6
11. Sample preparation for the investigation of the interaction between ssDNA and hBN flakes in different solvent environments 7
12. Schematic illustration for the fluorescence experiment 7
13. Solvent influence on the interaction between ssDNA and hBN 8
14. AFM images of DNA origami nanopores before hBN NP integration 9
15. AFM images of DNA origami nanopores after hBN NP integration 10
16. Aggregation due to the edge staples of the DNA origami 11
17. Correlative FM-AFM sample preparation 12
18. Correlative FM-AFM based on fiducial beads 13
19. hBN aggregation AFM results 14
20. Intensity of the integrated quantum emitters on the DNA origami 15

1. Production of hBN nanoparticles

Commercially available ultrafine hBN powder with a lateral size of about 100 nm was purchased from Graphene Supermarket. First, 18 mg of hBN powder was soaked in liquid nitrogen. After 1 h, the cryogenic hBN powder was immediately dispersed in the solvent of IPA/H_2O at a volume ratio of 1:1. The hBN suspension was then sonicated for 4 h using a commercially available ultrasonic water bath. After the cryogenic-pretreated LPE, the solution was centrifuged at 6000 rpm for 30 minutes to separate the nanoparticles from larger particles and aggregates. To further remove the aggregates in the dispersions, the final supernatant went through a filter with pore size of 100 nm.

2. AFM sample preparation and imaging

To prepare the AFM samples of hBN nanoparticles, a 20 µL drop of the diluted solution of hBN nanoparticles was deposited on a freshly cleaved mica substrate. To ensure uniform particle distribution onto the substrate and mitigate the coffee ring effect during drying, the mica substrate was preheated to 100 °C before deposition. The AFM images were recorded in air contact mode at room temperature using the Nanowizard JPK AFM with silicon tips.

To image the DNA origami and hBN-DNA origami complexes, a 2 µL drop of the diluted solution was deposited on a freshly cleaved mica substrate and left to absorb for 2 min and 5 min, respectively. 300 µL of the buffer (1×TAE, 10 mM $MgCl_2$) was added to the mica surface. The AFM images were recorded in liquid QI mode at room temperature using the Nanowizard JPK AFM with silicon tips.

To conduct the statistical analysis, several AFM images were obtained from separate locations across the mica surfaces to ensure the reproducibility of the results. All the image analyses were performed using the Gwyddion 2.0 software.

3. TEM sample preparation

hBN NPs were deposited onto glow-discharged carbon-coated grid (Quantifoil Micro tools GmbH, Germany), incubated for 2 minutes and then dried with ashless filter paper (VWR, France). Observations of the sample were carried out on a JEOL JEM1400 operating at 120 kV with a Matataki Flash sCMOS camera.

4. Microscopy setup

Fluorescent experiments were conducted on an inverted microscope (Nikon Instruments, Eclipse Ti2) with the Perfect Focus System. A water-immersion objective was used for the observation (Nikon Instruments, Plan Apochromat ×60/numerical aperture 1.2, H_2O). For the total internal reflection fluorescent (TIRF) microscopy, an oil-immersion objective was used (Nikon Instruments, Apo SR TIRF ×100/numerical aperture 1.49, oil). A laser box containing 3 different wavelengths (473 nm, 532 nm and 640 nm) was used for excitation. The fluorescent movies were collected by an sCMOS camera (Prime BSI Express) with an effective pixel size of 65 nm. The intensity of the laser source was set to 100 mW for hBN QE characterization under different wavelengths.

5. DNA origami design and assembly

The DNA origami was designed using caDNAno (v2.0). The two-dimensional DNA origami nanopore was designed as a single-layer rectangular structure composed of 30 helices, with a width of 60 nm and a length of 80 nm. The square hole in the center has a side length of 20 nm. 19 single-stranded DNA oligos were introduced on one side of the central cavity, each consisting of 30 adenine (A) deoxyribonucleotides.

The DNA staples were purchased from IDT Technologies, the scaffold strand M13mp18 (7249 bases) was purchased from Tilibit. The scaffold p7249 (M13mp18) at 10 nM was mixed with 50 nM of the staples in a buffer of 5 mM Tris-HCl, pH 8.0, 1 mM EDTA, 12.5 mM $MgCl_2$. The origami was subjected to a thermal annealing ramp: 90 ℃ to 20 ℃ -1 ℃ per every 5 minutes, then held at 20 °C. After the assembly, the DNA origami was purified by filtration (using Amicon Ultra-0.5 100K Centrifugal Filter) in the same buffer used during assembly to remove the excess staples.

6. MD simulation parameters

In this study, all molecular dynamics (MD) simulations were implemented using MaterialStudio. The initial establishment of molecular structures for adenine nucleobase ($C_5H_5N_5$), water (H_2O), and isopropyl alcohol (C_3H_7OH) was followed by an energy optimization process to attain their stable molecular conformations. One confined solution molecular model was created by dispersing $C_5H_5N_5$ molecules in H_2O/ C_3H_7OH hybrid solvent. In detail, there were 30 adenine nucleobase molecules, 200 H_2O and 200 C_3H_7OH molecules. Subsequently, the energy minimization and dynamic equilibrium processes were applied to the solution model under isothermal-isobaric (NPT) and canonical (NVT) ensembles using the DREIDING forcefield at 25°C and atmospheric pressure (101325 Pa). The time step and simulation time for each ensemble were set at 1fs and 500 ps, respectively. After MD simulations, the stable solution model exhibited a density around 0.8 g/cm^3, with cell lengths of 37.3 Å. Concurrently, the hexagonal boron nitride (hBN) unit cell underwent cleavage along the (1,1,1) surface, repeated 15 times in both X and Y directions to construct the hBN supercell layer. To investigate interactions between adenine nucleobase molecules and the hBN layer, a three-layer sandwich structure (hBN-solvent-hBN) was assembled. A dynamic simulation procedure utilizing the NVT ensemble was conducted for 500 ps at 25 °C to explore these interactions.

7. Agarose gel electrophoresis

For characterizing the formation of the DNA origami and DNA origami-hBN NPs complexes, a 1.5% agarose gel was cast with 0.5× TBE-Mg (5mM) buffer. The gel was run at 90 V in an ice-water bath for 70 min. Sybr safe stain was added with the sample for gel imaging. For band analysis, after gel electrophoresis, the gel was illuminated under a UV lamp (365 nm) to reveal the bands' location. For different solvent investigation in **Figure 4c**, the hBN NP in H_2O/IPA was first diluted two times in pure IPA, H_2O/IPA and pure H_2O, respectively. Then the diluted hBN NP was mixed with purified DNA origami with the volume ratio of 1:2, which leading to a final IPA volume ratio of 25%, 16.7% and 8.3%, respectively. The sample was incubated for 30 minutes before loading to the gel.

8. Fluorescent intensity traces under three illumination wavelengths

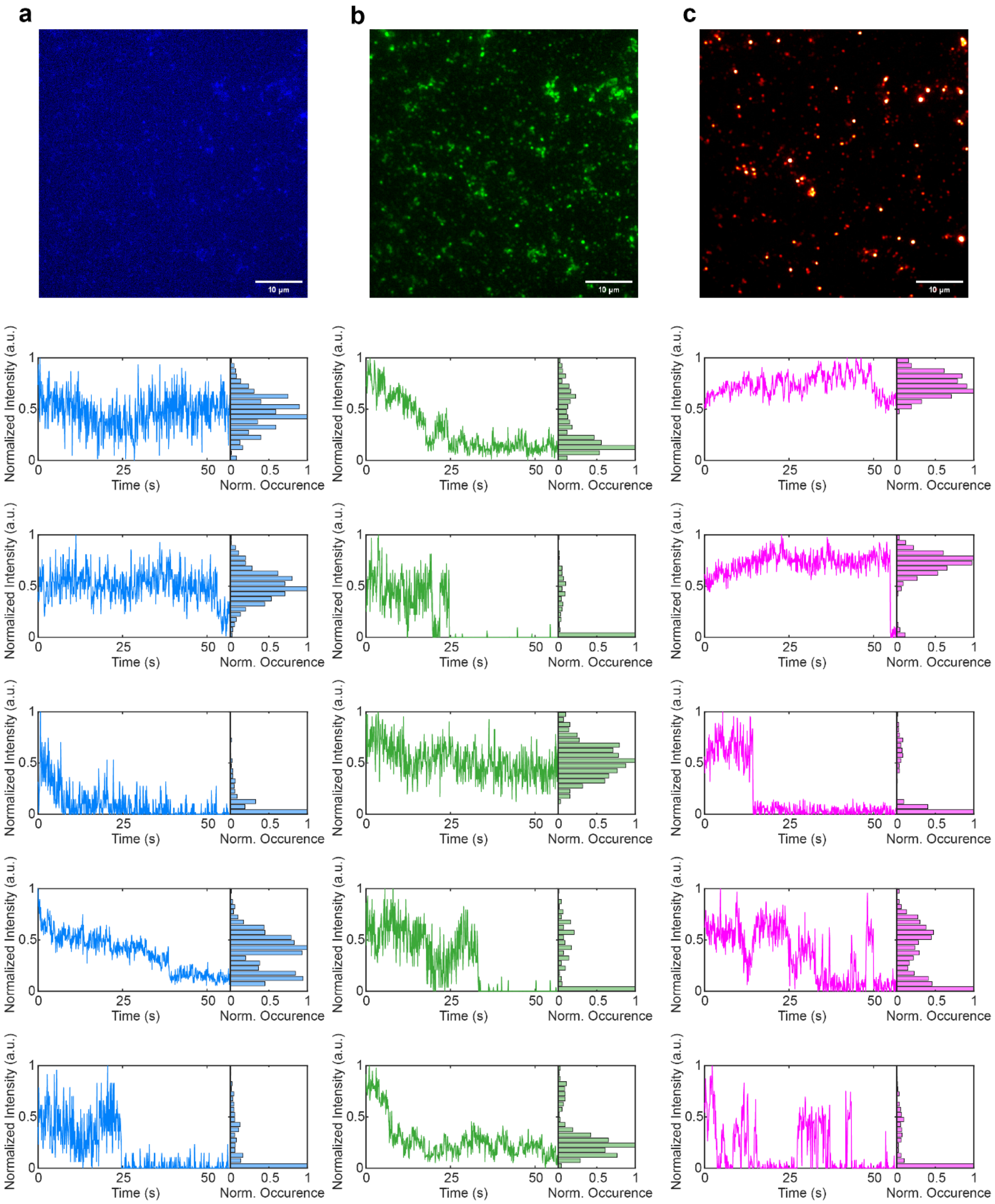

Figure S1. Fluorescence microscopy images and representative intensity traces of the hBN NPs under three excitations wavelengths (a: 473nm, b: 532nm and c: 640nm).

9. QE dynamics (stable/blinking) Classification

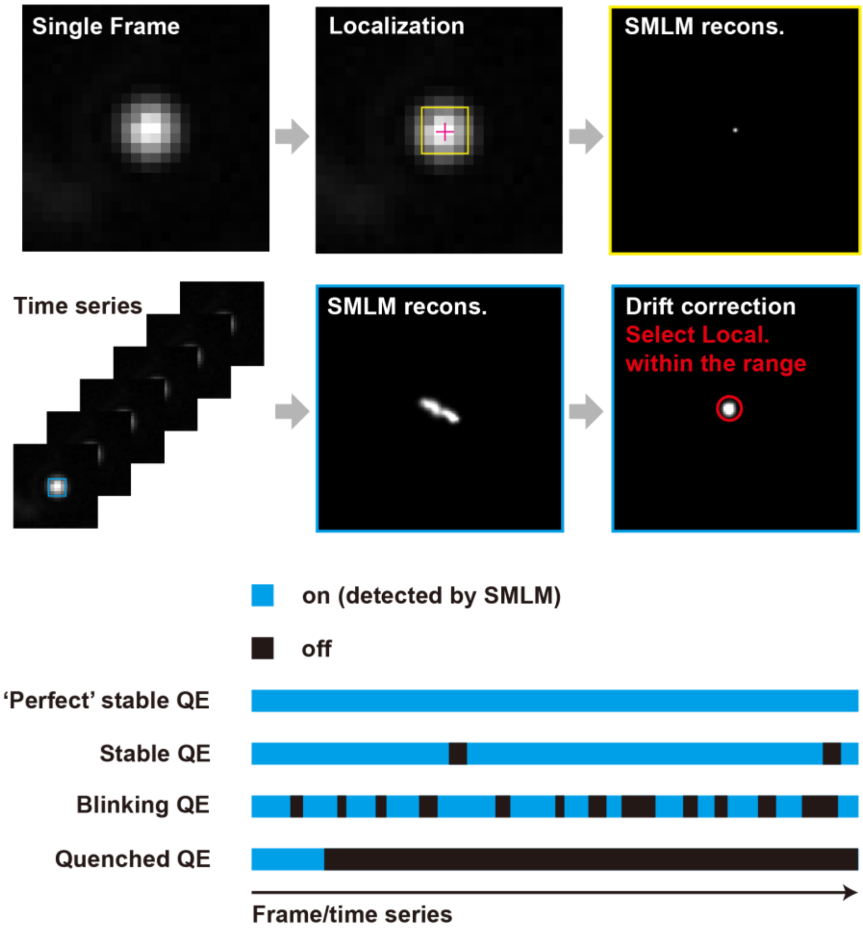

Figure S2. Workflow of the classification of the stable/blinking QEs under three illumination wavelengths: the SMLM reconstruction was finished in the ImageJ plugin ThunderSTORM, and RCC was used to do the drift correction. From the first to the last of the frame series, if a localization within the selective circle centred by the first localization is found, the QE at that frame is defined as 'on'. And the accumulated status in all the frames is used for the classification.

10. MD simulation results

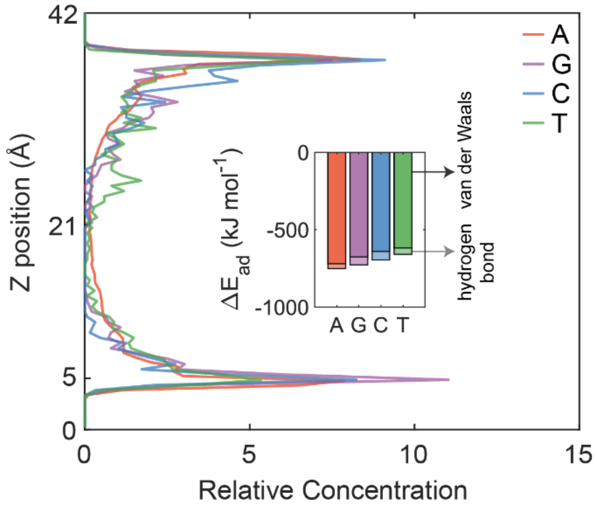

Figure S3. The MD setup was the same for each type of nucleobases. The concentration along the z direction reveals the absorption between the bases and the hBN flakes. The adsorption energy was inserted here to select the base candidate as the "sticky" part for the DNA origami.

11. Sample preparation for the investigation of the interaction between ssDNA and hBN flakes in different solvent environments

hBN flakes are transferred from transparent tapes to the clean coverslip. A PDMS liquid chamber is attached around the hBN flakes. Cy3B labelled ssDNA in different solvent is added to the liquid chamber, incubated for 5 minutes for the following fluorescence experiments.

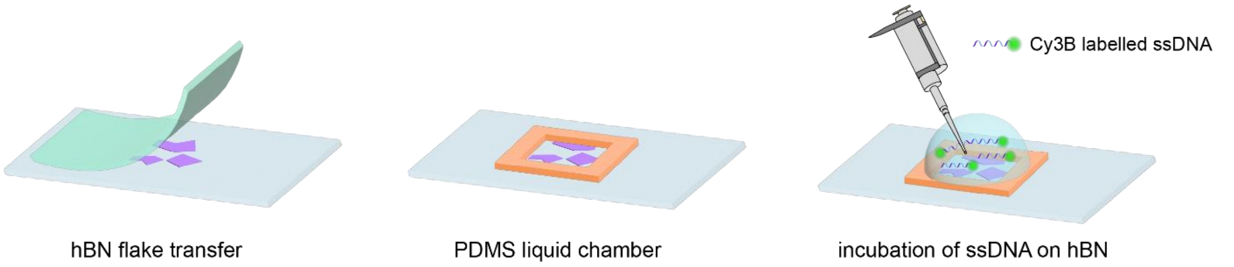

Figure S4. Schematic illustration for the sample preparation for the investigation of the interaction between ssDNA and hBN flakes in different solvent environments.

12. Schematic illustration for the fluorescence experiment

With the TIRF microscope, only the ssDNA attached to the hBN surface could be detected. In different liquid environments, higher affinity between ssDNA and hBN would lead to higher fluorescence spot density on the hBN flake. The lower affinity means more ssDNA diffuses randomly in the liquid, which will not excited by the TIRF evanescent field, leading to a lower spot density in the images.

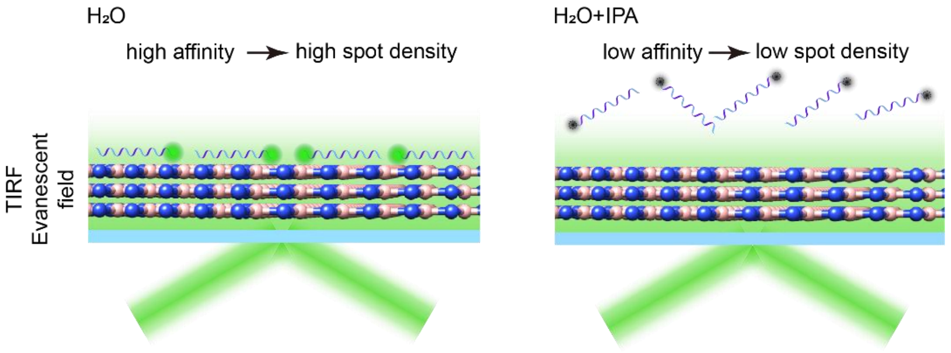

Figure S5. Schematic illustration of the fluorescence imaging experiment showing where differences in ssDNA to hBN affects the single-molecule spot density.

13. Solvent influence on the interaction between ssDNA and hBN

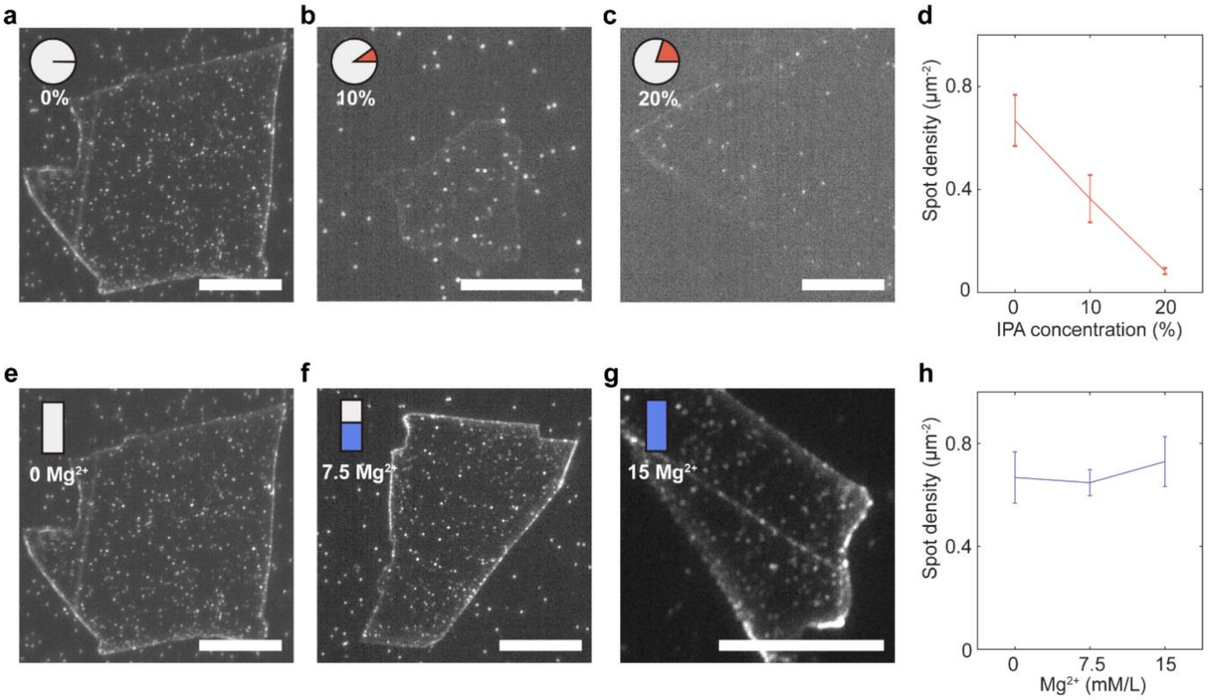

Figure S6. Single molecule measurements of the distribution of the ssDNA on hBN flakes. (a-c) Fluorescence images of Cy3B labelled ssDNA on the hBN flakes in liquid environment with different IPA volume concentrations. From (a) to (c), the IPA volume concentration is 0%, 10%, and 20% from three different hBN flakes. (d) Average spot density over 1500 frames per concentration, which shows a decreasing trend with increasing IPA concentration. (e-g) Fluorescence images of Cy3B labelled ssDNA on the hBN flakes in liquid environment with different magnesium concentration. From (a) to (c), the Mg^{2+} concentration is 0 mM, 7.5 mM, and 15 mM. (d) Average spot density over 1500 frames per concentration, which does not show a significant change with different salt conditions.

14. AFM images of DNA origami nanopores before hBN NP integration

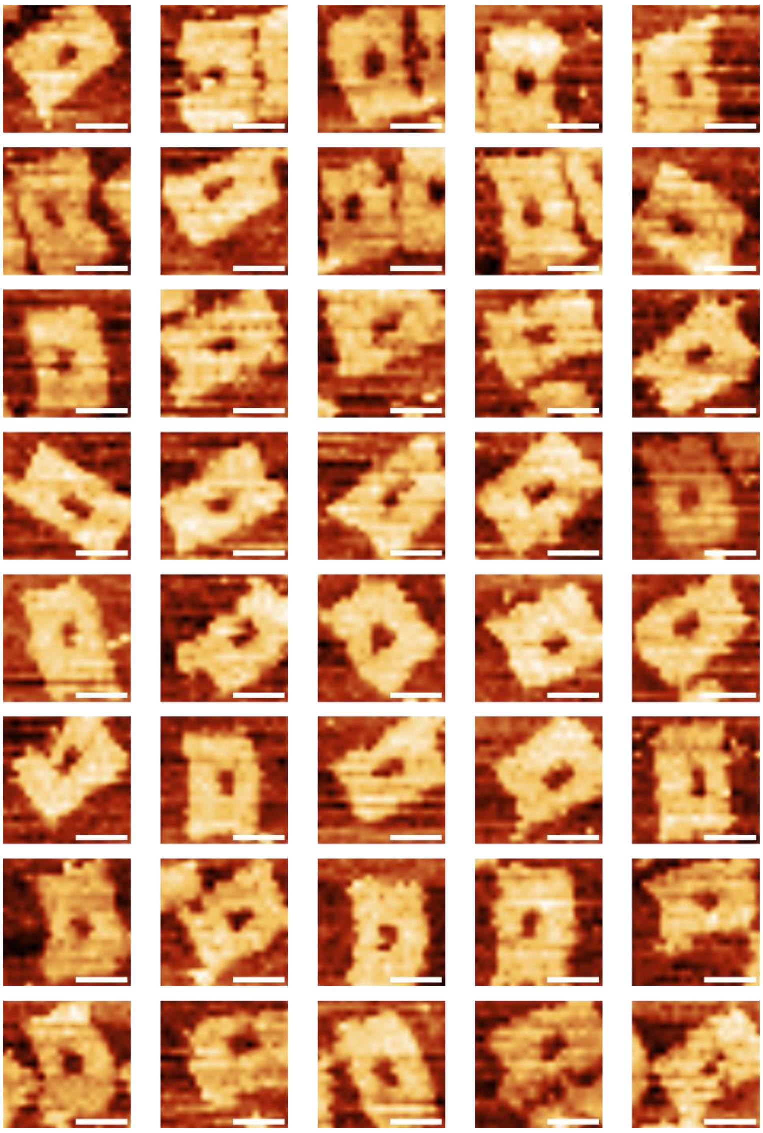

Figure S7. Topography images of bare DNA origami plates on mica surface. (Scale bar: 50 nm).

15. AFM images of DNA origami nanopores after hBN NP integration

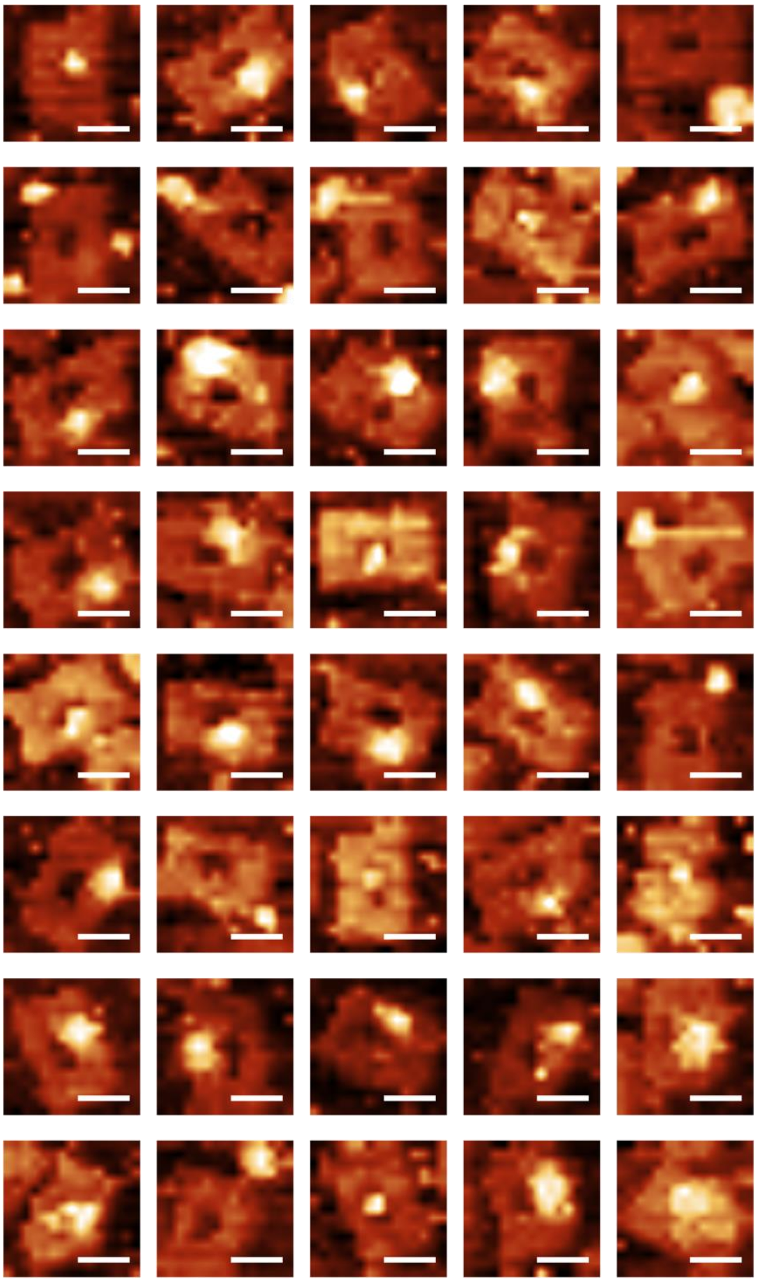

Figure S8. Topography images of hBN NPs-DNA origami complex on mica surface. (Scale bar: 50 nm).

16. Aggregation due to the edge staples of the DNA origami

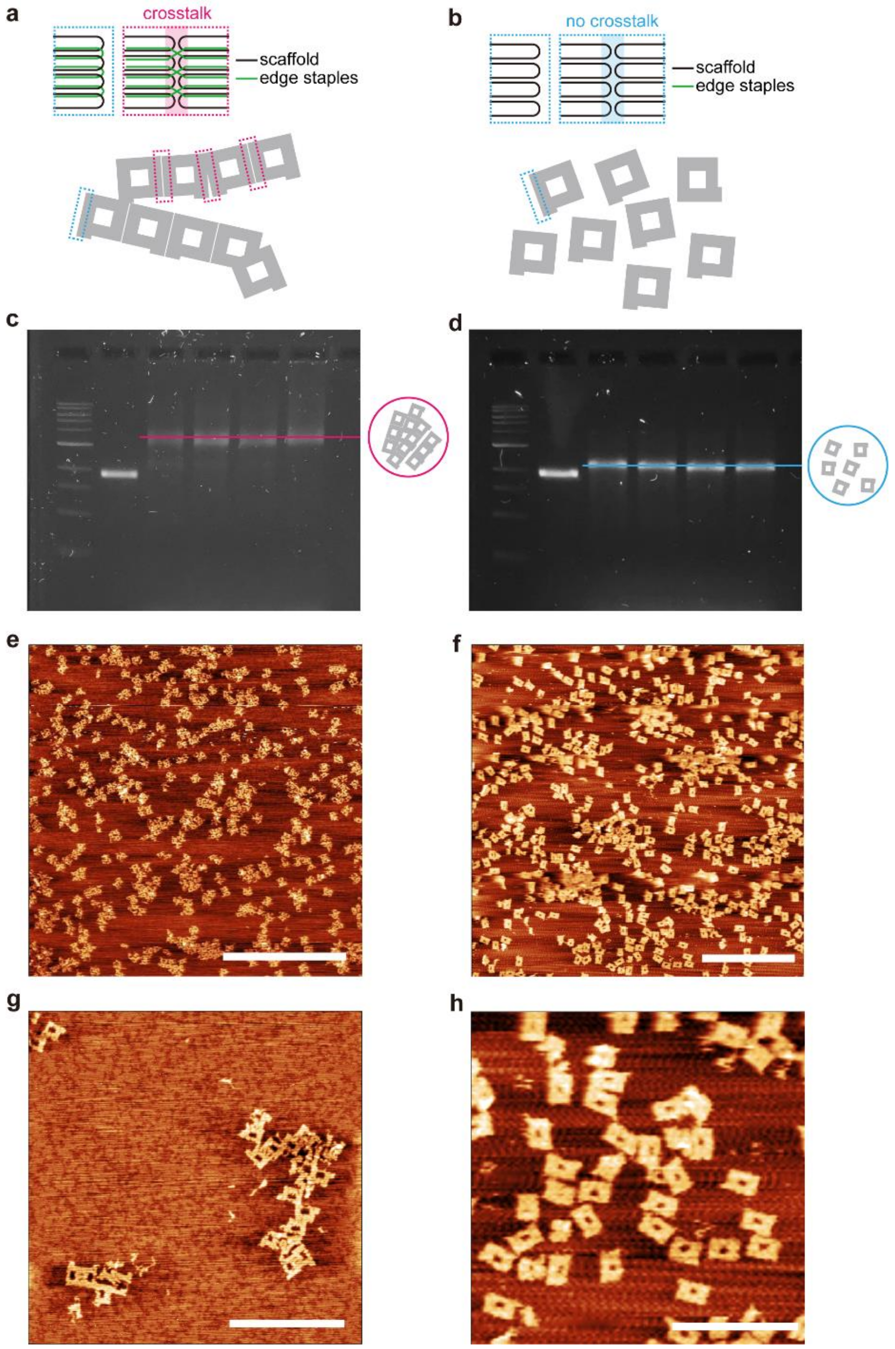

Figure S9. Comparison of the assembled DNA origami with/without edge staples. (a) Schematic illustration of the aggregation due to the edge staples. The staples on the edge lead to some crosstalk between different origamis. (b) The aggregation could be alleviated by removing the edge staples. (c)(d) Gel electrophoresis experiment for the DNA origami through same assembly protocol with/without edge staples, where large aggregation due to the edge staples could be clearly distinguished in (c). For each gel, from left to right is DNA ladder, M13mp18 scaffold, DNA origami assembled in different Mg^{2+} concentrations: 7.5 mM, 10 mM, 12.5 Mm and 15mM. (e)(g) AFM measurement for large area/zoom in for the aggregated sample. Scale bar: 2 µm and 500 nm, respectively. (f)(h) AFM measurement for large area/zoom in for the well separated sample by removing the edge staples. Scale bar: 1 µm and 400 nm, respectively.

17. Correlative FM-AFM sample preparation

Figure S10 shows the correlative fluorescence microscopy (FM) and atomic force microscopy (AFM) sample preparation protocol. Considering that the working distance of the objective lens of the fluorescence microscope is more limited than that of the AFM, and to avoid the influence of refractory cracks in the mica substrate, we replaced the substrate of the sample from mica to poly-lysine-coated thin coverslips (170 µm thickness). Coverslips were washed with acetone and isopropanol in successive rinses and sonicated for 3 min each. To ensure uniform distribution of polylysine on the slides, we used two separate methods: the first was to drop 7 µl of a 0.01% mass fraction solution of polylysine onto the coverslips after 10 min of oxygen plasma cleaning of the washed coverslips, curing for 3 minutes and then rinsing with deionised water and blow-drying with a nitrogen gun. The second was to add polylysine solution directly onto the cleaned coverslip, and at the same time rotate the other cleaned coverslip by 90° and place it on the coverslip with the solution, thus forcing the solution to be evenly distributed in the intersecting position of the two coverslips, and similarly rinsing it with deionised water and drying it with a nitrogen gas gun after 3 minutes of curing. No significant difference was found between the above two methods. A suspension of microspheres in water was added to the poly-lysine coated coverslip, and a drop of the sample of the hBN-DNA hybrid sample was added after the evaporation of the solvent in the suspension of microspheres.

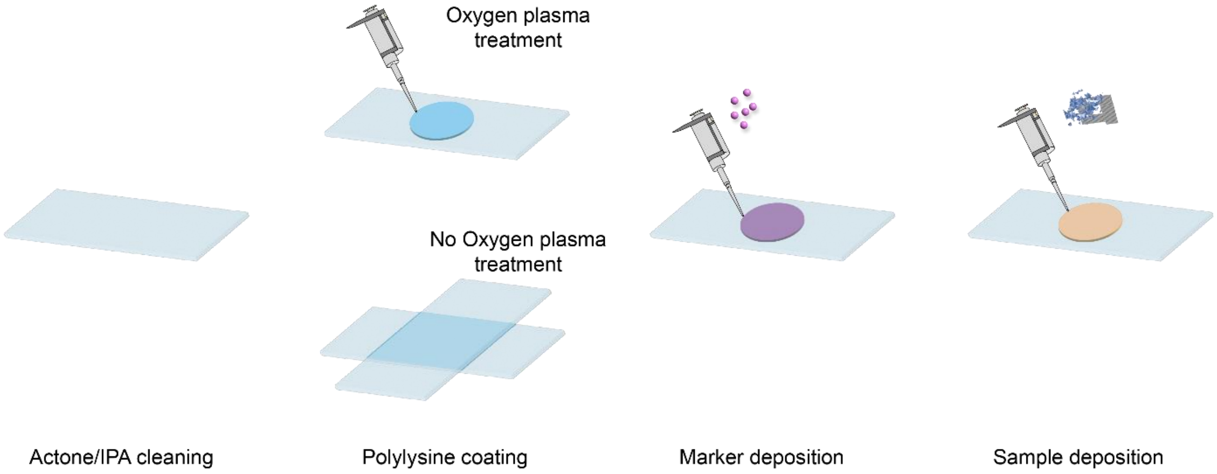

Figure S10. Steps for correlative FM-AFM sample preparation**.**

18. Correlative FM-AFM based on fiducial beads

Mesh calibration protocol.

1) To insert the AFM measurement into the microscopy images, we calibrated the scanning direction of the AFM tip and used it to build a scanning grid, which facilitated us to couple the fluorescence microscope and AFM images by inserting the AFM result into the scanning grid according to the head position (**Figure S11a-c**).

2) Correlation of AFM with FM (**Figure S11d-g**): In the AFM, we first adjusted the focal plane to the centre of the microsphere and captured the image as reference for correlation. Under the fluorescence microscope, we first adjusted the imaging focal plane to the center of the microspheres and captured the image formed due to microsphere refraction as reference for correlation. With two images from different instruments focused on the centre of the microspheres, we can set the centres of the microspheres as the reference point to calculate the angular and displacement offsets of the two instruments and thus correlate them.

3) Single molecule localization microscopy (SMLM) reconstruction (**Figure S11h-k**): The focal plane was adjusted to the plane where the QEs are located to collect the image sequence for SMLM reconstruction. The AFM and SMLM were correlated and the transparency of the SMLM was adjusted based on the intensity for clear visualization.

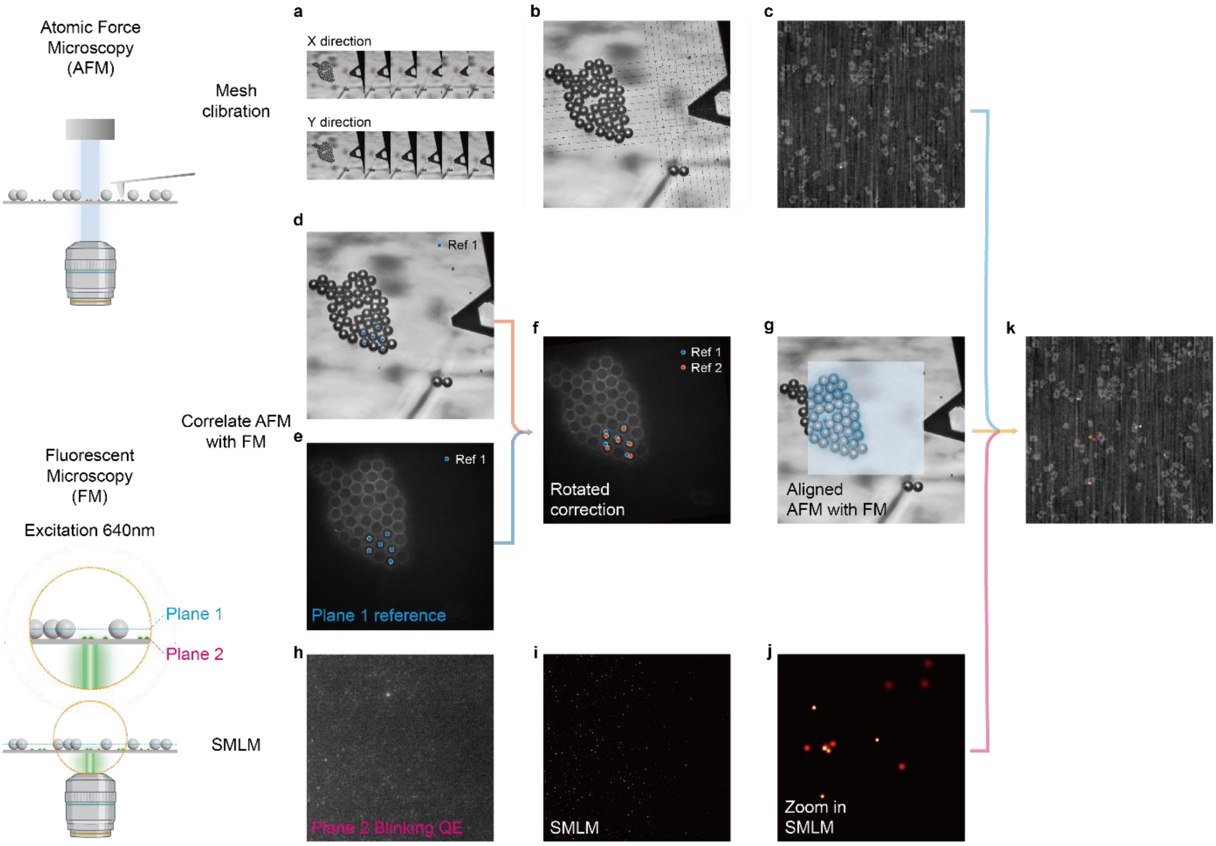

Figure S11. Correlative FM-AFM based on fiducial beads.

19. hBN aggregation AFM results

It is quite common to find some areas with hBN aggregation on the sample surface, as shown in **Figure S12**. These areas were excluded from the analysis.

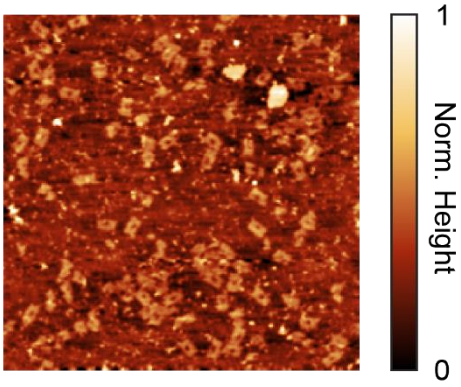

Figure S12. AFM image of an area with hBN aggregation and DNA origami.

20. Intensity of the integrated quantum emitters on the DNA origami

Figure S13 shows the correlative AFM-SMLM measurement for the hBN functionalized DNA origami. In the first stage, even with some fluctuation, all four emitters could be recognized by the SMLM algorithm. In the second stage, one of the emitters is quenched. In the final stage, only two emitters could be detected. Overall, the fluorescence properties of the quantum emitters aligns well with the previous measurement of bare hBN quantum emitters, which proves the stability of the hBN quantum emitters in physiological buffers and when attached to biological components.

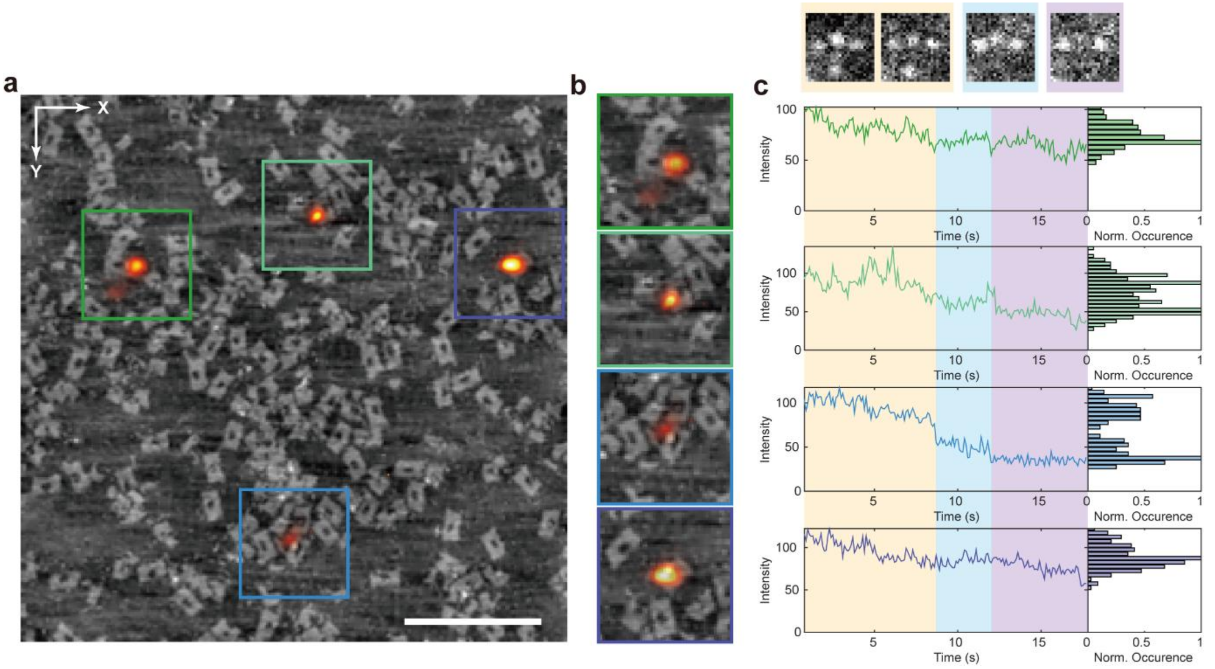

Figure S13. (a) Correlative AFM-SMLM images for the sample. (b) Zoom in on the representative samples. (c) Intensity fluctuation of the hBN quantum emitters after the integration on the DNA origami.
